# 

**Published:** 1850-06

**Authors:** 


### NOTICE TO CORRESPONDENTS.

Communications and Books for notice should be addressed to the Editor, care of Messrs. Lindsay & Blakiston.

Letters, &c., connected with the *business affairs* of the Journal should be addressed to the Publishers.

Papers for publication must be received *before* the 20th of the month, or they cannot appear in the forthcoming number.

The following Journals have been received in exchange:

The Boston Medical and Surgical Journal. (Weekly, Boston.)
Buffalo Journal.
New York Journal of Medicine.
Medical News.
Western Lancet.
Ohio Medical and Surgical Journal.
New Orleans Medical and Surgical Journal.
Southern Medical and Surgical Journal.
Charleston Medical Journal and Review.
Western Jourual.
St. Louis Medical and Surgical Journal.
North-Western Medical and Surgical Journal.
The British American Journal of Medical and Physical Science. (Montreal.)
The London Lancet. (Weekly, London.)
The Medical Times. (Weekly, London.)
Dublin Medical Press.
Provincial Medical and Surgical Journal.
British and Foreign Medico-Chirurgical Review.
London Journal of Medicine.
London Medical Gazette.
London Medical Examiner.
Pharmaceutical Journal.

---

The following works have also been received for notice:

The Diseases of the Interior Valley of North America. By D. Drake, M. D., &c.

Remarks on Cholera. By E. J. Coxe, M. D., New Orleans.

Accommodation of the eye to distance. By W. C. Wallace, M. D. From the Author.

A Memoir on Stricture of the Urethra. By J. P. Mettauer, M. D., LL. D. From the Author.

Essays on the Puerperal Fever, by F. Churchill, M. D. From Messrs. Lea & Blanchard.

Report on the Cholera in Boston. From Dr. Clarke.

Ship Fever; being the Fisk fund Prize Essay for 1849. By H. G. Clarke, M. D.

Taylor's Medical Jurisprudence. From Messrs. Lea & Blanchard.

Essay on Alcohol; by Wm. B. Carpenter, M. D. From the same.

Experiments on Warming and Ventilating Hospitals. By T. S. Kirkbride, M. D.

Essay on the Opium Trade. By N. Allen, M. D.

Proceedings of the Medical Society of North Carolina.

Transactions of the Medical Society of the State of New York.

Historical Sketch of the state of Medicine in the American Colonies. By J. B. Beck, M. D.

---

Communications have been received from :—

W. J. Reese, M. D., Alabama.
William Waters, M. D., Fredericktown, Md.
Charles Sutherland, M. D., Philadelphia.
S. E. McKinley, M. D., Williamsport, Tenn.

---

The foreign correspondents of the Examiner will please direct their Exchanges and other communications to the care of Mr. Charles J. Skeet, 27 King William St., Charing Cross, London, or Mr. H. Bossange, 21 Bis, Quai Voltaire, Paris.

